# STM Study of the Initial Stage of Gold Intercalation of Graphene on Ir(111)

**DOI:** 10.3390/ma16103833

**Published:** 2023-05-19

**Authors:** Vesna Mikšić Trontl, Ivan Jedovnicki, Petar Pervan

**Affiliations:** Institute of Physics, 10000 Zagreb, Croatiapervan@ifs.hr (P.P.)

**Keywords:** graphene, 2D structure, gold, intercalation, scanning tunnelling microscopy (STM)

## Abstract

In this paper, we present a study of the sub-monolayer gold intercalation of graphene on Ir(111) using scanning tunnelling microscopy (STM). We found that Au islands grow following different kinetics than growth on Ir(111) without graphene. Graphene appears to increase the mobility of Au atoms by shifting the growth kinetics of Au islands from dendritic to a more compact shape. Graphene on top of intercalated gold exhibits a moiré superstructure, with parameters significantly different from graphene on Au(111) but almost identical to graphene on Ir(111). The intercalated Au monolayer shows a quasi-herringbone reconstruction with similar structural parameters as on Au(111).

## 1. Introduction

The rationale for the strong interest in the research of graphene interaction with metallic surfaces stems from (i) the ease of synthesizing monolayer and multilayer graphene on a large scale and with outstanding quality, and (ii) the possibility of studying almost free-standing graphene as a result of weakly bound graphene on some metal surfaces [1,2,3]. While the first property is important for technological applications (e.g., graphene-based touch screens), the second allows researchers to study many basic physical phenomena of pristine-like graphene. Graphene on (111) faces of Ag, Pt and Ir is known for a weak interaction [4,5,6], while Ni(111) [7], Rh(111) [8] and Ru(0001) [9] are metal surfaces that are suitable substrates for the fabrication of high-structural-quality graphene [10], but at the same time are very poor in preserving the graphene electronic structure due to the strong perturbation by hybridization with *d*-bands of underlying metals [1]. In order to restore the nearly intrinsic electronic structure of the graphene, the intercalation process [11] is used to place different elements, such as Ag [12], Cu [13,14], and alkali metals [15,16], at the graphene–substrate interface.

Gold surfaces are known for their weak interaction with graphene [17], but also for the difficulties of synthesizing it due to the lack of hydrocarbon decomposition activity. For this reason, different strategies have been devised to create graphene layers on top of gold substrate surfaces [5,18]. The very simple approach is to grow graphene on one of the (111) faces of transition metals (e.g., Ni or Ir) and subsequently intercalate it with gold [5,19]. Among these, the Au intercalated Gr/Ni(111) is the most studied system, experimentally [17,19] and theoretically [20,21]. As has been demonstrated by several experiments, a graphene band initially destroyed due to strong hybridization with 3*d* Ni bands is entirely recovered upon gold intercalation [22]. Gold has been intercalated under graphene grown on Ir(111), where graphene islands have been studied [5,23]. The interest in graphene on gold also originates from the fact that it possesses a low level of charge transfer (around 6.2 × 10^11^ holes cm^−2^), which is lower only for graphene on Ir(111). Such a charge transfer leads to a shift of the Dirac point of 0.24 eV above the Fermi level [5,18,23]. In addition, there are virtually no interactions between graphene and Au bands close to the Fermi level, which makes this system exceptionally suited for investigations of quasi-free-standing graphene. The argument for the weak graphene–gold interaction is corroborated by the persistence of the gold-distinct ‘herringbone’ surface reconstruction characteristic for Au(111) [24]. Namely, the appearance of the herringbone structure at Au surfaces is the result of a rather subtle energy balance which is, due to the very weak interaction, obviously not affected by the presence of a graphene overlayer [18,23].

Graphene intercalated by gold on Ir(111) has been studied only for thick gold films which exhibit moiré super-structures with a periodicity of 17.0 Å due to the 14.7% lattice mismatch between graphene and Au(111) [5].

In this paper, we present an STM study of graphene on Ir(111) intercalated by a sub-monolayer coverage of gold. We studied the structure of the intercalated gold in terms of the possible influence of graphene on its interaction with the underlying iridium surface.

## 2. Materials and Methods

The STM and low energy electron diffraction (LEED) experiments were performed in an ultra-high vacuum (UHV) setup operating at a base pressure of 5 × 10^−10^ mbar. The setup was used for the characterization of a clean, graphene-covered Ir(111) surface and Au intercalated graphene on Ir(111). The STM measurements were performed on Aarhus STM (SPECS) at room temperature (R.T.). The STM images were recorded while scanning the surface by an electrochemically etched tungsten tip, in constant current mode, with a bias voltage applied to the sample. The STM was calibrated by measurements on the HOPG sample and the STM images were processed with WSxM software 5.0. The Ir(111) sample was a crystal 6 mm in diameter, of 99.99% purity, polished with roughness <0.03 μm and with an orientation accuracy <0.1°. The sample was heated by e-beam heating using a hot filament at a negative potential near the grounded sample. The sample temperature was calibrated by the C-type thermocouple attached to the sample with respect to the heating time and power used and, additionally, values were measured by a K type thermocouple attached to the sample plate. The C-type thermocouple was then removed from the sample to enable multiple graphene preparations and transfer from the sample preparation manipulator to STM.

The Ir(111) sample was cleaned by using cycles consisting of 50 min of 1 keV argon-ion sputtering followed by 10–25 min of annealing in oxygen partial pressure of 1 × 10^−7^ mbar at 1100 K, and a final 5–15 min annealing in UHV at 1150 K. After five such cycles, typically a sharp LEED pattern and large-scale STM images confirmed a clean surface.

The graphene monolayer on Ir(111) was prepared by a temperature programmed growth cycle (TPG, room temperature ethylene exposure 6 × 10^−8^ mbar for 60 s and flash to 1400 K) followed by chemical vapour deposition (CVD, 6 × 10^−8^ mbar of ethylene for 300 s while the sample held at 1150 K). This TPG + CVD procedure growth led to a uniform orientation of graphene (referred to as Gr/Ir(111)) with the lattice aligned to the substrate lattice (R0) and at full monolayer coverage. Gold was deposited by resistive heating of a tungsten basket filled with pure gold (purity: 99.999%) heated by the direct current through the W wires, while the sample was kept at the R.T. The intercalation was carried on immediately after Au deposition by sample post-annealing at 800–900 K.

## 3. Results and Discussion

**Figure 1:**
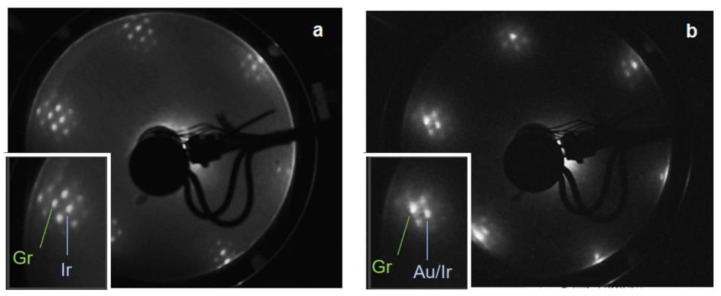
LEED patterns of (**a**) graphene on Ir(111) and (**b**) gold-intercalated graphene on the same surface. The insets show the details of the LEED patterns with the main spots indicated.

We first characterised Gr/Ir(111) before and after Au intercalation by means of LEED. Figure 1a shows the characteristic (1 × 1) LEED pattern of Gr/Ir(111) of hexagonal symmetry, with additional spots associated with the moiré superstructure. The LEED pattern shows a predominantly R0 graphene orientation, and no contribution from R30 is observed [25]. The intercalation of Au enhances the graphene spots with moiré superstructure spots still visible, reflecting the conservation of long-range moiré periodicity (see Figure 1b).

As can be evidenced from the inset in Figure 1b, in contrast to graphene on Au(111) we could not see any diffraction signatures of herringbone superstructure [23]. The STM results presented in the following provide a reasonable explanation for the lack of a herringbone superstructure fingerprint.

**Figure 2:**
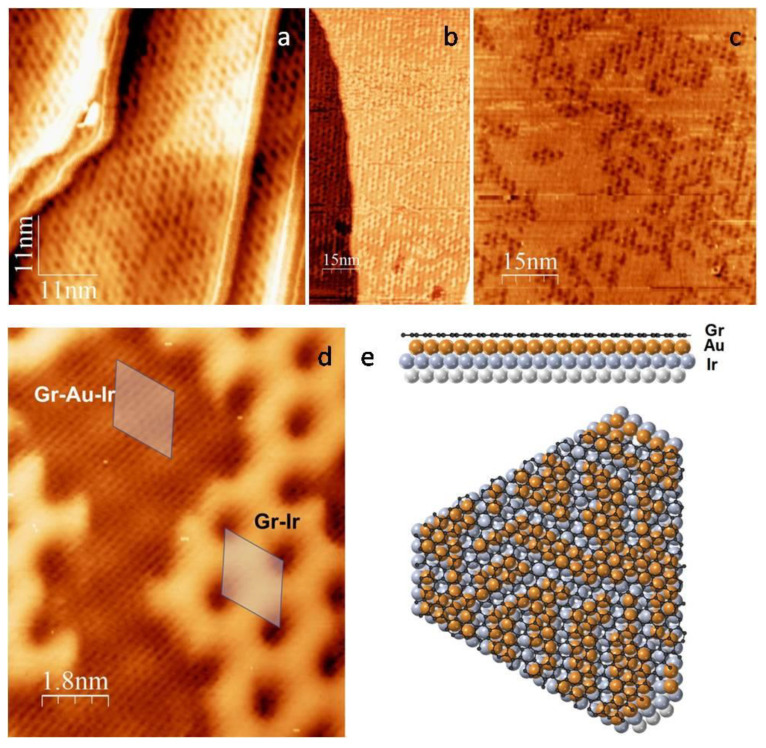
STM images of graphene on Ir(111), partly intercalated by Au for different Au coverage (**a**–**c**). (**a**) 0.4 ML; (**b**) 0.5 ML; (**c**) 0.8 ML. (**d**) STM image showing the details of intercalated (Gr-Au-Ir) and non-intercalated (Gr-Ir) areas. The scanning parameters are (**a**) −140 mV, 0.29 nA; (**b**) 464 mV, 0.85 nA; (**c**) 540 mV, 0.78 nA; and (**d**) 94 mV, 1.31 nA. (**e**) Approximate schematic of the atomic layers showing side- and top-view of graphene on Ir(111) partly intercalated by Au. Pseudomorphic growth of Au on Ir(111) is assumed.

The intercalation of graphene by Au produced a visible impact on the STM images of the Gr/Ir(111) system (Figure 2). They exhibited two types of areas (patterns). Figure 2d shows an atomic resolution image of partly intercalated graphene.

The bright areas with enhanced contrast and clear hexagonal structure correspond to the moiré superstructure of non-intercalated graphene. The area characterised by weak moiré modulation is associated with the intercalated graphene regions. From Figure 2d, we can identify the moiré unit cell of intercalated graphene, which is of the same size as the non-intercalated. In the following, we elaborate on this statement more quantitatively. Figure 2a,b clearly suggests that areas of gold-intercalated graphene, in the shape of rugged islands, are homogeneously distributed over the surface.

**Figure 3:**
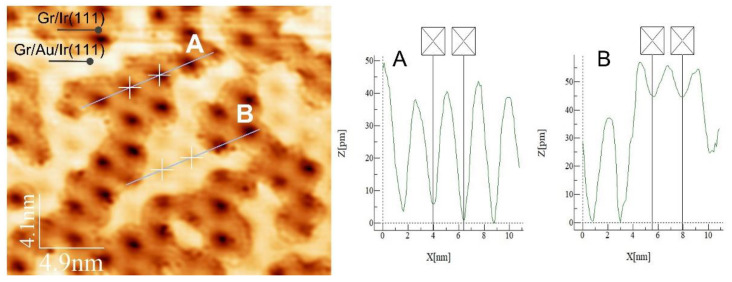
STM image of graphene on Ir(111) partly intercalated by Au and line profiles across non-intercalated (line A) and intercalated (line B) areas. Scanning parameters: 302 mV, 0.49 nA.

A comparison of the images in Figure 2a,b and Figure 1 in Refs. [26,27] provides compelling evidence that graphene introduces a significant difference in the Au growth mode on Ir(111). Namely, as has been shown, Au grows on bare Ir(111), having a dendritic shape with a triangular envelope [26,27]. In contrast, Au intercalates on Ir(111), forming islands with no preferential shape percolating at coverage below 0.5 ML (see Figure 3). This suggest a somewhat increased mobility of Au atoms when intercalated compared to the case when they are adsorbed, which is rather surprising. However, the shape of intercalated Au islands appears to still be away from the thermodynamic equilibrium [28]. One should bear in mind that the shape of metal islands can be strongly influenced by residual gas [29]. However, the influence of the residual gas on the growth kinetics of intercalated atoms is extremely unlikely, as the graphene behaves as a highly protective mesh [30].

We measured the moiré periodicity across the area with intercalated and non-intercalated graphene (see Figure 3). The lines in the STM image and the corresponding line profiles marked as A and B were associated with non-intercalated and intercalated graphene, respectively. From profiles A and B, we can draw two conclusions: (i) the moiré periodicity of the Au intercalated graphene equals the periodicity of the non-intercalated graphene on Ir(111)—25 Å; and (ii) the corrugation of the intercalated graphene is substantially smaller (0.12 Å) than the corrugation of bare graphene (0.35 Å), suggesting a reduced interaction of the graphene with gold compared to iridium. At this point, it is interesting to compare these parameters for graphene on the Au(111) surface [5], where the moiré periodicity was found to be 17 Å. The corrugation of graphene on Au(111) was measured to be a tenth of the corrugation on Gr/Ir(111)—just 0.04 Å.

**Figure 4:**
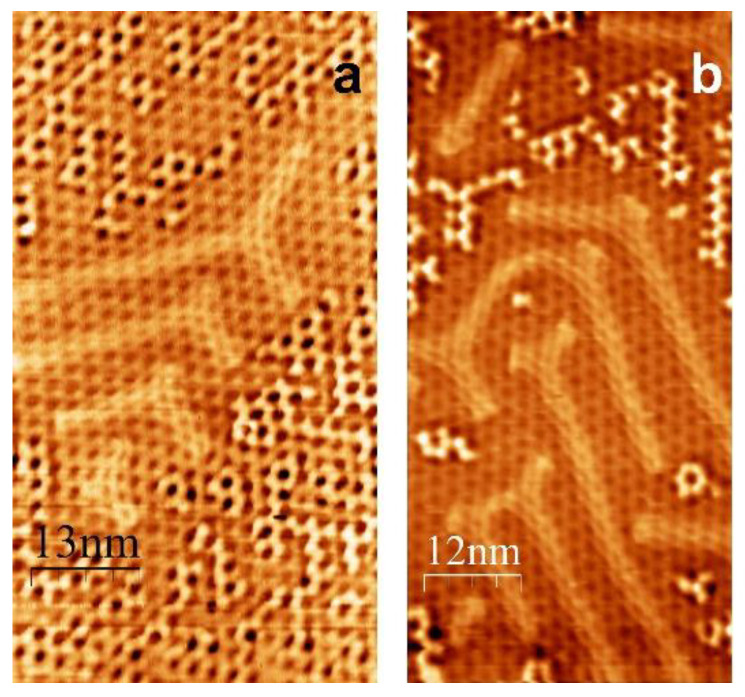
STM image of the partly Au-intercalated Gr/Ir(111), showing herringbone reconstruction of intercalated gold. Scanning parameters: (**a**) −180 mV, 1.52 nA; (**b**) −149 mV, 3.47 nA.

STM images, given in Figure 4, show areas which are dominantly intercalated by gold. These images clearly show that the intercalated gold exhibits quasi-herringbone reconstruction, and the observed superstructure is the result of the interference of moiré and the quasi-herringbone pattern. It has been already established that a (111) gold surface of thick Au films and bulk Au crystals covered by graphene displays this distinctive reconstruction [5,18,23], typical for Au(111) [31]. In contrast, for the sub-monolayer Au-intercalated gr/Ir(111) presented here, due to the remnants of the non-intercalated graphene, the pattern of herringbone reconstruction is not as fully developed as on the Au(111) [31], i.e., there is no long-range periodicity in herringbone reconstruction which could be reflected in LEED. However, it seems that the reconstruction that took place in Au between gr and Ir(111) has similar parameters as on the bulk Au(111) [31,32] or thick Au film [23] for, in both cases, the periodicity of herringbone pattern is found to be around 60 Å and corrugation around 0.17 Å [5].

The fact that the gold monolayer between gr and Ir(111) exhibits the same reconstruction with similar structural parameters as the Au(111) surface layer indicates that the structure of the gold overlayer is dominated by the inter-atomic interaction over the adsorbate–substrate one. However, the herringbone pattern is not registered in the Au monolayer on Ir(111), suggesting a noticeable influence of graphene on the observed reconstruction. On the other hand, graphene on Au(111) had no impact on the reconstruction of the surface layer [18]. Having in mind the subtlety of the long-range elastic interactions which drive this reconstruction [33], Wofford et al. [18] used the absence of the graphene influence on the reconstruction itself as an argument for the level to which graphene is decoupled from the gold surface. The influence of graphene on the morphology of Au islands is even more puzzling in the intercalated phase.

Regarding the metals weakly interacting with graphene, intercalation can change the growth mode, e.g., from cluster to smooth films (ref. [13]). However, there is rather scarce evidence of graphene-induced reconstruction in systems where graphene, weakly interacting with monolayers or sub-monolayers of metals, induced their reconstruction [34,35]. As reported by Jolie et al. [34], Ag-intercalating graphene flakes on Ir(111) exhibit a complicated aperiodic structure. It appears that the emergence of multiple rotational domains, combined with a strong dependence on the local strain, is responsible for the absence of the short-range pattern order. The Gr/Pt/Pt(111) system, studied by Halle et al. [35], showed a modification to the Pt(111) reconstruction upon the intercalation of Pt. The modification was ascribed to the change in the adsorption energy landscape for Pt on Pt(111).

To correctly interpret the emergence of the quasi-herringbone pattern in sub-monolayer Au intercalated between graphene and Ir(111), a theoretical investigation of electron transfer and the potential energy landscape of the system is required.

**Figure 5:**
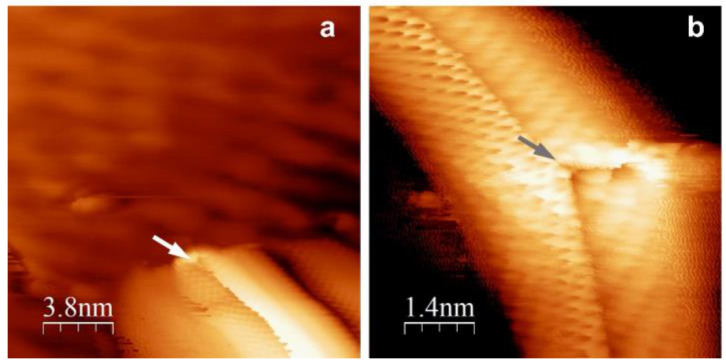
STM images of graphene wrinkles. Arrows are pointing to the defect sites. Scanning parameters: (**a**) 251 mV, 2.2 nA; (**b**) −320 mV, 2.6 nA.

Finally, Figure 5a,b shows STM images, partially with atomic resolution, of graphene wrinkles which are established as an important factor in the intercalation process. Additionally, wrinkles could be considered as nanochannels which improve atom diffusion and mass transport of the metal intercalant between graphene and substrate [36].

The graphene grows on Ir(111) in a continuous layer over macroscopic distances and wrinkles, together with multi-atom vacancies are regarded as preferential penetration channels for graphene intercalants which interact weakly with graphene [37,38,39,40]. We believe that gold intercalates at the graphene–Ir(111) interface by the same intercalation mechanism.

## 4. Conclusions

In conclusion, we have studied the sub-monolayer gold intercalation of graphene on Ir(111). We found that Au islands grow following different kinetics to the growth on Ir(111) without graphene. It appears as if graphene increases the mobility of Au atoms, shifting the growth kinetics of Au islands from dendritic towards a more compact shape. Graphene on top of intercalated gold shows a moiré superstructure with parameters significantly different to graphene on Au(111), but almost identical to graphene on Ir(111). The intercalated Au monolayer exhibits well-known herringbone reconstruction, with similar structural parameters as on Au(111). The explanation of the emergence of this reconstruction requires a theoretical investigation of electron transfer and the potential energy landscape of the system, and is beyond the scope of the present work. Nevertheless, the observed effects can provoke interest in further theoretical calculations, e.g., the DFT of the intercalated gr/Au/Ir(111).

## Data Availability

Reasonable requests for data can be addressed to V.M.T.
